# Trepid and Intrepid Travelers

**DOI:** 10.3201/eid1108.050534

**Published:** 2005-08

**Authors:** Eric V. Granowitz

**Affiliations:** * Baystate Medical Center and Tufts University School of Medicine, Springfield, Massachusetts, USA

**Keywords:** Trepd and Intrepid travelers, Africa, martha’s vineyard, travel

When I proposed travel to Africa for our honeymoon, Edy wrinkled her nose. "Why?" she asked.

"It will be wonderful!" I exclaimed. "The people are different. The animals are different. Even the countryside is different. It will be a fantastic getaway. We'll have a great time. Can you think of anyplace better to go?"

"Martha's Vineyard," she answered. "Someplace nearby. I don't want adventure. I just want to spend time with you." Sensing my disappointment, she added, "Why not go to Africa for our first anniversary?"

After the wedding and a wonderful trip to Martha's Vineyard, we relocated. I joined a new infectious diseases division, and Edy worked as a self-employed, real estate title examiner. We began planning our safari to Kenya and Tanzania. Edy would see the flamingos of Lake Nakuru, I the wildlife on the Serengeti Plain.

In the months preceding our departure, a New York Times article on political violence in coastal Kenya precipitated anxious telephone calls to the State Department and the US embassy in Nairobi. We ordered DEET, permethrin, a water purification device, and oral rehydration salts. I cringed a little when I wrote a nonrefundable check for the trip. Edy cringed a lot when we received our yellow fever, meningococcal, and hepatitis A immunizations. Mary Jo, the nurse in our travelers' health clinic, went over a list of dietary precautions. When Edy heard from a friend about mefloquine's neuropsychiatric side effects, I could not palliate her fears. Mary Jo was able to counsel her.

The week before departure, Edy sprayed our clothes with permethrin. Another call to the State Department confirmed no recurrence of political violence. We began taking mefloquine. Because I had a history of anaphylaxis to bee venom, I had a prescription filled for an epinephrine autoinjector.

In Nairobi (Figure) we were greeted by the touring company. A representative drove us through shanty-lined roads to an elegant country club. Next morning, we met our tour group: Noel, an Australian with a quick smile and no previous travel experience, who was embarking on a yearlong trip around the world; Bob, a broad-shouldered Mormon in a sailing T-shirt, who resembled "Mr. Clean"; Paula and Dan, a wealthy couple from Southern California. For someone with an impressive travel résumé, Dan appeared anxious. The source of his anxiety was Paula. While her eyes were filled with life, her body was not. Her ill-fitting wig, pale complexion, and unsure gait reminded me of patients on the oncology ward at the hospital. To avoid being identified as the tour physician, I introduced myself as assistant professor at a New England university.

**Figure:**
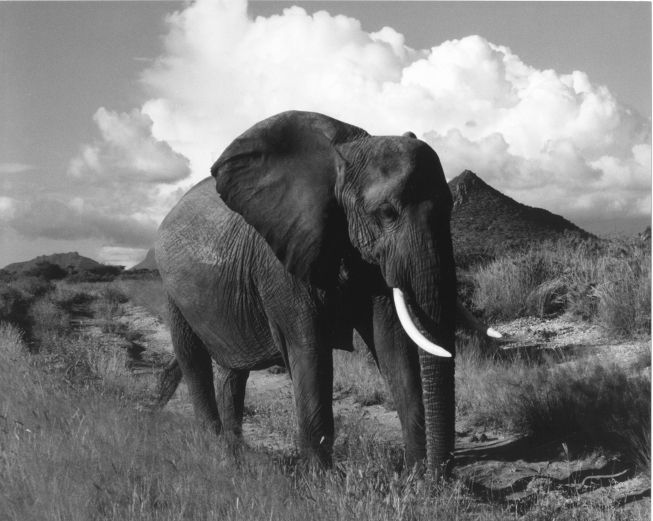
This savannah elephant (*Loxodonta africana*) did not ask the author for medical advice during his vacation. Photograph courtesy of Edyce Winokur

While the rest of us tried to get over jetlag, Edy and Dan braved a thunderstorm to take a shuttle downtown. The next morning, I awoke to find Edy with her hands folded over her abdomen and a worried expression on her face. "Ice cream sundae," she admitted, when I asked what she had eaten in Nairobi. After a dose of ciprofloxacin and loperamide, Edy made her way to the jeep. Her eyelids drooped when the guide announced the 8-hour drive to our destination. Although Edy made it to the game park uneventfully, her dining partner was not as fortunate. Despite antimicrobial treatment for probable salmonellosis, Dan had fever and explosive diarrhea. Noel experienced recurrent emesis and diarrhea, probably from brushing his teeth with tap water. Forced by these circumstances to reveal my identity as an infectious disease specialist, I now became "the doctor." Dan and Noel recovered uneventfully, but my vacation changed irrevocably.

Most of our meals were now punctuated by, "Can I eat this?" The query was directed at me. "Because this is a 5-star hotel," I responded, "that's probably okay. But generally, if the food hasn't been cooked, boiled, or peeled, don't eat it." The follow-up question was, "Would you eat it?" Being conservative, I smiled and shook my head when they pointed to the salad.

When our tour group grew, the questions multiplied. People asked about vaccinations, although almost everyone had been properly immunized. There was a case of doxycycline-associated photosensitivity in a woman who did not apply sunscreen. Two people stopped malaria chemoprophylaxis because of perceived adverse effects. Another woman told me she was glad a doctor was on the trip so she didn't have to be so careful with food. I was changing, from wanting to help to becoming resentful. My companions seemed unwilling to follow simple precautions, yet expected care when their "indiscretions" made them ill.

A new companion, Lester, got travelers' diarrhea. I suggested bottled water and beverages without ice cubes. Before I could even ask, Edy reached into her knapsack and gave Lester ciprofloxacin and loperamide. That evening, I heard Lester's wife screaming my name. I arrived to find Edy's patient unconscious and held upright by his friends. After we moved Lester into the Trendelenburg position, he quickly regained consciousness. The next morning, Lester was off for a ride in a hot air balloon.

Six months before our trip, Paula had undergone a stem cell transplant for non-Hodgkin's lymphoma. She had since experienced recurrent emesis and anemia unresponsive to erythropoietin, now complicated by lightheadedness and near syncope. When Dan asked if they should abandon the tour, I recommended they return home. They remained on safari. Eventually, hematocrit of 13 at an altitude of 5,000 ft proved too much for Paula. She was flown to the hospital for emergency transfusion.

Finally, our trip concluded, Edy and I settled in for the flight home. My thoughts were a menagerie of lions, elephants, rhinoceroses, giraffes, oryxes, ostriches, and vultures. I relived the experience of watching a pack of hyenas tear the entrails out of a wild-eyed wildebeest. In spite of every effort to free itself, the wildebeest could not escape. Similarly, even on vacation, I could not escape being a doctor. My personal life had once again been compromised by my professional responsibilities.

I fell asleep. Some time later, Edy roused me with a nudge. "I was proud of the way you took care of people," she declared. This was the first time Edy had seen me practice medicine, and she had enjoyed watching me attend to our group. Her comments reminded me that caring for others was a privilege.

Before I could express these thoughts, Edy continued, "This was a great vacation. Where do you want to go next year? Thailand? It would be wonderful. The people are different. The animals are different. Even the countryside is different. It will be a fantastic getaway. We'll have a great time. Can you think of anyplace better to go?"

"Martha's Vineyard," I said.

